# Auto-Administered Photobiomodulation on Diabetic Leg Ulcers Treatment: A New Way to Manage It?

**DOI:** 10.1155/2020/7428472

**Published:** 2020-05-20

**Authors:** Elisabetta Merigo, Lixin Tan, Zengyi Zhao, Jean-Paul Rocca, Carlo Fornaini

**Affiliations:** ^1^Faculty of Dentistry, University “Côte d'Azur”, 5 Avenue du 22ème BCA, 06357 Nice, France; ^2^Department of Diabetes, 2nd Hospital, Gongnong Road 425, Shijiazhuang, Hebei Province, China; ^3^Department of Stomatology, 2nd Hospital, Gongnong Road 425, Shijiazhuang, Hebei Province, China; ^4^GAEM Group of Applied Electro Magnetics, Department of Engineering and Architecture, University of Parma, Viale G. P. Usberti 181/A-43124, Parma, Italy

## Abstract

**Background:**

Peripheral arterial disease is a dramatic consequence of an uncontrolled diabetic condition causing an increase of morbidity and mortality and its treatment is currently medical or surgical, finally requiring, in the 7–20% of cases, major or minor amputation. Photobiomodulation therapy (PBM) is a laser treatment used in medicine, thanks to its ability to stimulate the wound healing, the acceleration of inflammatory process, and the modulation of pain. Recently, the self-administration of the treatment has been suggested for different purposes in medicine and dentistry with a great number of advantages and no side effects.

**Methods:**

A 84-year-old woman affected by diabetes type 2 and positive for diabetes complications had diagnosis for an ulcerative lesion of 1 cm diameter on her right leg and started a treatment of the lesion applying the B-Cure Laser Pro (Erika Carmel, Haifa, Israel) on her own with a fluence per minute of 3.2 J/cm^2^ for 2 sessions of 15 minutes by cutaneous application.

**Results:**

After a week of treatment, the ulcer dried and crusted, finally providing complete healing after 30 days of treatment.

**Conclusion:**

With this short case report, we think to add a further contribution by suggesting this kind of treatment for successful management of the leg ulcers in diabetic patients.

## 1. Introduction

Peripheral arterial disease is one of the main consequences of an uncontrolled diabetic condition and also the main cause of ulcers leading to the so-called diabetic foot ulcers (DFU) and diabetic leg ulcers (DLU), which are a complication affecting about 15% of the diabetic patients [1, 2] and causing an increase of morbidity and mortality [3]. The treatment of this disease is currently medical, by means of a broad-spectrum antibiotic therapy, or surgical, finally requiring, in the 7–20% of cases, major amputation (leg or thigh) or minor amputation (toes or foot), being both procedures at risk of mortality [3–5].

Photobiomodulation (PBM) therapy is a laser treatment used in medicine for different purposes, thanks to its ability to stimulate, by means of a photochemical interaction with the tissues, the wound healing, the acceleration of inflammatory process, and the modulation of pain [6].

Its use in the treatment of diabetic foot ulcers has been recently reported by several studies: Li et al., in their meta-analysis paper, described the usefulness of PBM in treating diabetic foot ulcers in order to improve healing rate, reduce ulcer diameter, and relieve pain, by using wavelengths of 400–904 nm with a fluence of 2–10 J/cm^2^ [7], while Maya et al. suggested the use of a wavelength of 632.8 nm and a fluence of 3.1 J/cm^2^ [8]. Mathur et al. highlighted the use of 660 nm laser at a fluence of 3 J/cm^2^ with similar positive results [9].

Tantawy et al. compared, in addition to conventional therapy, He-Ne laser (wavelength of 632 nm) PBM performed during 90 seconds with a fluence of 5 J/cm^2^ and 904 nm diode PBM performed at 25 Hz for 90 seconds with a fluence of 6 J/cm^2^ and found a significant reduction in ulcers surface without statistically significant difference between the 2 laser devices used [10].

Recently, the “at-home PBM” treatment has been suggested for different purposes in medicine and dentistry. It is based on self-administration of the treatment by the patient with a great number of advantages and no side effects [11–13].

The aim of this work is to show the possibility to use, in the treatment of ulcers in diabetic foot/leg, this new laser device recently proposed in the market.

## 2. Case Report

**Figure 1:**
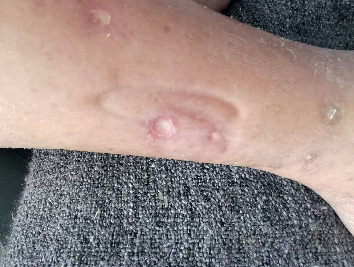
First clinical observation of ulcerative lesions on the right leg.

A 84-year-old Chinese woman, affected by diabetes type 2 from 32 years and positive for diabetes complications such as eye disease, nephropathy, neuropathy, and vascular disease, was evaluated for a routine control by her diabetologist. She was under insulin treatment and her blood sugar balance was acceptable. During the visit, the doctor noticed, probably due to the itchy skin, the presence of painful ulcerative lesions (the biggest one of 1 cm diameter) on the lowest part of the right leg (Figure 1), which the patient described as appeared for at least 3 weeks.

**Figure 2:**
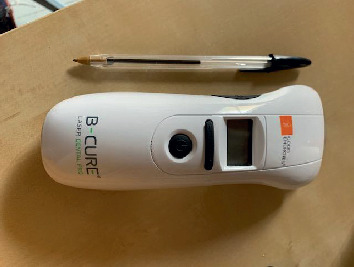
The device used for the treatment of the case, B-Cure Laser Pro (Erika Carmel, Haifa, Israel), emitting in the infrared spectrum (808 nm).

The same day, the patient, after giving her informed consent about the treatment and also allowing the publication of the pictures, started the treatment on the lesions applying B-Cure Laser Pro (Erika Carmel, Haifa, Israel) on her own. This device (Figure 2) emits in the infrared spectrum (808 nm) with a green LED aiming beam indicating the irradiation area, which is an elliptic shape with the two axes measuring 45 and 10 mm for a total surface of 4.5 cm^2^. It was not necessary, for the patients, to wear protective glasses because the appliance used is classified as class I device by the American National Standard Institute.

When applied in contact to the target tissue, outperformed plastic allows to maintain the beam at a distance of 10 mm. In this way, it is able to deliver to the tissues an output power of 250 mW (55.5 mW/cm^2^) emitted in micropulses with a frequency of 15 kHz, for an energy per minute of 14.4 Joules and a fluence per minute of 3.2 J/cm^2^.

**Figure 3:**
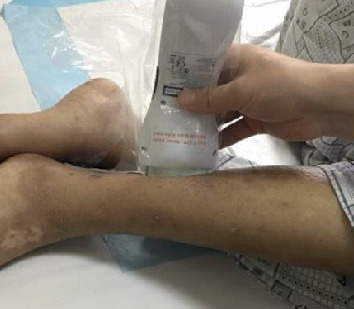
At-home application of laser B-Cure on ulcerative lesion: a simple transparent plastic bag was used to wrap the laser and to maintain sterility.

**Figure 4:**
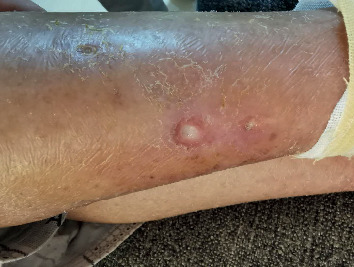
Clinical appearance after a week of treatment: the smallest ulcers (upper and right lower) dried and crusted, and the largest one (lower) achieved a significant improvement.

**Figure 5:**
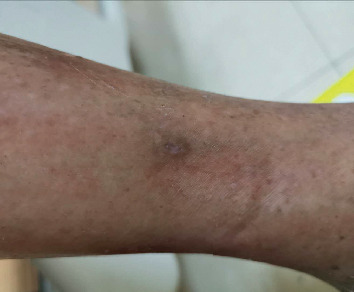
Complete healing of all the lesions after 30 days of the laser treatment.

Treatment was daily performed for 2 sessions of 15 minutes (total fluence 48 J/cm^2^) by cutaneous application (Figure 3); after a week of treatment, the smallest ulcers (upper and right lower) dried and crusted (Figure 4) and the largest one (lower) had a significant improvement. Finally, all the ulcers got completed healed after 30 days of treatment (Figure 5).

## 3. Discussion

The use of self-administered laser PBM in this case of ulcerative lesions related to diabetes disease gained an interesting result in terms of healing. Results described for this case report are related to mechanisms of laser PBM as the promotion of physiological effects such as keratinocyte and fibroblast proliferation, maturation and migration, collagen synthesis and deposition, activation of macrophages, and overall in DFU/DLU endothelial cell proliferation, neoangiogenesis and revascularization [6]. The choice of the wavelength of 808 nm was supported by previous reports in the literature describing evidence for the stimulation effects particularly on endothelial cells. Amaroli et al. in 2019 described effects in wound healing repair for treatment performed with an 808 nm laser at a fluence of 60 J/cm^2^ [14].

Diabetes is a disease with a high impact on a population in terms of prevalence, incidence, and morbidity, and diabetic foot ulcers is one of its most important consequences with very severe difficulties in its management and treatment and with devastating consequences for the patients in terms of quality of life.

Diabetic foot ulcers and diabetic leg ulcers are complications with a high impact in terms of morbidity, mortality, and healthcare costs, involving, as reported by the International Diabetes Federation, between 9.1 and 26.1 million people with a 5% mortality in the first 12 months and a 42% mortality within 5 years [14].

Actually, standard care includes local wound care with surgical debridement, dressings promoting a moist wound environment, wound off-loading, vascular assessment, treatment of active infection, and glycemic control with an estimated cost of US $176 billion (report 2012) but unfortunately also with a 20% of patients have unhealed at 1 year [15].

The protocol described may obtain good results in terms of pain control and patient compliance. PBM therapy is a noninvasive therapy already described as useful for the management of this complication; unfortunately, the limiting factor of this kind of treatment consists on the needing, for the patients, to go to the therapist at least twice/three times weekly for treatments of some minutes, and this factor may negatively influence the compliance of the patient toward the therapy.

Due to the reduced size and the belonging to the class I laser according to the ANSI classification, of the “at-home PBM” laser device, it may be self-administered at home by the patient, and this represents a great and innovative possibility to manage the treatment of this kind of diseases. The availability on the market of these new, cheap, small, and usable at home by the patients themselves PBM appliances might be included in standard therapy for this kind of problem, giving the possibility to the patients to receive PBM treatment also daily; having the patient only a time setting as part of the device, the danger of over treating is reduced.

## 4. Conclusion

At-home PBM therapy is a new chapter of the laser medicine, which was born when new small appliances able to be used by patients themselves appeared in the market. More and more fields of utilization were proposed in these last years, and by means of this short case report, we think to add a further contribution by suggesting this kind of treatment for successful management of the foot ulcers in diabetic patients and encouraging research development to improve the strength of evidence for this application.
